# Antioxidant-Based Therapy Reduces Early-Stage Intestinal Ischemia-Reperfusion Injury in Rats

**DOI:** 10.3390/antiox10060853

**Published:** 2021-05-27

**Authors:** Gaizka Gutiérrez-Sánchez, Ignacio García-Alonso, Jorge Gutiérrez Sáenz de Santa María, Ana Alonso-Varona, Borja Herrero de la Parte

**Affiliations:** 1 Department of Surgery and Radiology and Physical Medicine, University of The Basque Country, ES48940 Leioa, Biscay, Spain; gaizka_gutierrez@hotmail.es (G.G.-S.); ignacio.galonso@ehu.eus (I.G.-A.); jorge.gssm@gmail.com (J.G.S.d.S.M.); 2 Interventional Radiology Research Group, Biocruces Bizkaia Health Research Institute, ES48903 Barakaldo, Biscay, Spain; 3 Department of Cell Biology and Histology, University of The Basque Country, ES48940 Leioa, Biscay, Spain; ana.alonsovarona@ehu.eus

**Keywords:** antioxidant treatment, intestinal ischemia-reperfusion injury, allopurinol, nitroindazole, animal model

## Abstract

Intestinal ischemia-reperfusion injury (i-IRI) is a rare disorder with a high mortality rate, resulting from the loss of blood flow to an intestinal segment. Most of the damage is triggered by the restoration of flow and the arrival of cytokines and reactive oxygen species (ROS), among others. Inactivation of these molecules before tissue reperfusion could reduce intestinal damage. The aim of this work was to analyze the preventive effect of allopurinol and nitroindazole on intestinal mucosal damage after i-IRI. Wag/RijHsd rats were subjected to i-IRI by clamping the superior mesenteric artery (for 1 or 2 h) followed by a 30 min period of reperfusion. Histopathological intestinal damage (HID) was assessed by microscopic examination of histological sections obtained from injured intestine. HID was increased by almost 20% by doubling the ischemia time (from 1 to 2 h). Nitroindazole reduced HID in both the 1 and 2 h period of ischemia by approximately 30% and 60%, respectively (*p* < 0.001). Our preliminary results demonstrate that nitroindazole has a preventive/protective effect against tissue damage in the early stages of i-IRI. However, to better understand the molecular mechanisms underlying this phenomenon, further studies are needed.

## 1. Introduction

Intestinal ischemia-reperfusion injury (i-IRI) is an uncommon disorder that affects 0.09 to 0.2% of hospitalized patients. It involves decreased intestinal blood flow followed by a local inflammatory response that leads to necrosis of the intestinal wall, and in the absence of correct and quick management, may lead to patient death (the mortality rate is reported to be as high as 90%). The acute onset results from an abrupt and spontaneous interruption of intestinal blood flow due to occlusion of a venous or arterial vessel, or to non-occlusive causes (NOMI). NOMI comprises approximately 20% of cases of intestinal ischemia, which are commonly related to superior mesenteric artery (SMA) vasoconstriction or poor cardiac performance that triggers hypoperfusion of the intestine [1,2,3,4].

This blood deprivation hinders the aerobic metabolism of enterocytes, compromising mitochondrial oxidative phosphorylation, forcing the cells to perform anaerobic metabolism for energy production [5]. Moreover, the inhibition of the Na^+^/K^+^-ATPase pump activity alters the transmembrane potential and the electrolyte balance of the intra- and extracellular media, generating edema that is of special interest at the time of reperfusion [6]. In addition, the different intermediate active metabolites generated during the different cellular reactions cannot be secreted into the systemic circulation for renal excretion, thus, they accumulate inside the cells and contribute to cell damage and death [5]. However, the greatest damage during i-IRI does not occur in the ischemia period, but rather, during reperfusion of the ischemic tissue with the arrival of oxygen and the action of cytokines, the complement system, reactive oxygen species (ROS), neutrophils and the alteration of capillary permeability, which, in addition to promoting expansion to the rest of the organism, leads to bacterial translocation [7].

**Figure 1:**
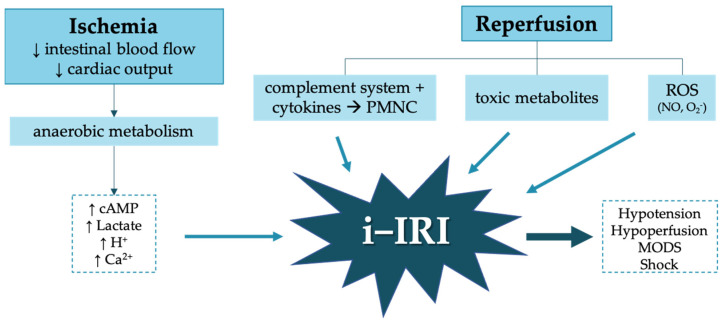
Pathophysiology of mesenteric ischemia reperfusion syndrome (i-IRI). cAMP (cyclic adenosine monophosphate), PMNC (polymorphonuclear cells), ROS (reactive oxygen species). The upward pointing arrow (↑) signifies increase, and The downward pointing arrow (↓) signifies decrease.

Damage at the systemic level as a consequence of i-IRI is mainly mediated by toxic metabolites produced during the ischemia period and the affinity and high reactivity of ROS for other tissues. These toxic metabolites generate an imbalance between the intracellular and extracellular environment, which triggers fluid sequestration at the extracellular level, the creation of a third-space and a decrease in effective circulating volume. This manifests clinically as hypotension, shock, or even systemic inflammatory response syndrome (SIRS), and multiple organ dysfunction syndrome (MODS), which also perpetuates the low cardiac output situation [8] (Figure 1).

The therapeutic management of NOMI-related i-IRI must be individualized, based on the underlying precipitating cause. The main therapeutic approaches include fluid resuscitation, optimization of cardiac output, and elimination of vasopressors; furthermore, vasodilators or antispasmodics may also be used. In those patients in whom ischemia has resulted in severe infarction of an intestinal segment, surgical resection of the affected portion is necessary; however, when this resection involves an important segment of intestine, it can trigger short bowel syndrome [4].

These approaches are mainly aimed at restoring intestinal flow as soon as possible [3]. However, another interesting and complementary approach is the management of oxidative stress damage by decreasing/inhibiting the action of the ROS. Xanthine oxidase (XO) and nitric oxide synthase (NOS) play a key role in ROS production. There are compounds such as allopurinol, which is used in the management of chronic gout that inhibit XO, preventing the generation of ROS by this pathway. The prophylactically administration of this compound in animal models or in clinical trials of cardiac, hepatic or renal ischemia-reperfusion injury (IRI) has demonstrated its protective effect, acting at the level of vascular permeability, polymorphonuclear cells infiltration, bacterial translocation, chemokine signaling, motility and mortality derived from this situation [9,10,11,12,13,14]. On the other hand, nitroindazole, a compound of the imidazole and indazole family, inhibits the neuronal nitric oxide synthase isoform (nNOS), which induces a decrease in nitric oxide (NO) synthesis during the early stages of reperfusion with no alterations observed during the period of ischemia or late reperfusion [15,16]. Moreover, it has been shown that this molecule contributes to protein degradation and cell damage during IRI [17]. Therefore, it is expected that blocking or decreasing the activity of XO and NOS could prevent or minimize reperfusion-associated damage in the organism as a whole.

This study analyzes the individualized effect of the prophylactic administration of these two compounds, due to their recognized antioxidant capacity, as therapeutic tools for the treatment of i-IRI by analyzing the response at the anatomopathological level.

## 2. Materials and Methods

All procedures were carried out in accordance with current legislation and were approved by the Animal Experimentation Ethics Committee (CEEA) (reference M20/2019/207) and the Biological Agents Research Ethics Committee (CEIAB) of the University of The Basque Country (reference M30/2019/208).

Sixty male 3-months-old WAG/RijHsd rats were induced to develop i-IRI following a 1 or 2 h period of ischemia and 30 min of reperfusion, and were randomized into 10 groups (Table 1). The animals were maintained in 12 h light/dark cycles with food and water ad libitum. Another 6 animals were used as a control.

### 2.1. Surgical Procedure

**Figure 2:**
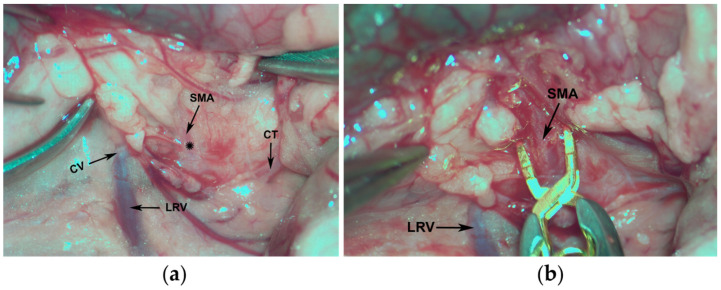
View of the surgical field: (**a**) anatomical location of the main structures: CV (cava vein), LRV (left renal vein), CT (celiac trunk), and SMA (superior mesenteric artery), the asterisk indicates the site of placement of the microvascular clip; (**b**) detail of the dissected SMA with the microvascular clip.

Under isoflurane anesthesia (1.5%), a middle laparotomy was performed to locate the SMA (Figure 2a). Briefly, part of the small intestine was pulled out over moistened gauze and the superior mesenteric artery was dissected, and then clamped with a Yasargil microvascular clamp (Figure 2b). Once the absence of arterial pulse in the mesenteric region was observed, the bowel was reintroduced and the abdominal muscles were sutured with a running suture and the skin was sutured with single stitches. Prior to the animal’s recovery, a single dose of meloxicam (2 mg/kg, sc) was administered.

When the ischemia period was over (1 or 2 h), the animals were re-anesthetized, the laparotomy was reopened, and the Yasargil clip was removed. The wound was closed as previously described and 30 min of reperfusion time was allowed.

### 2.2. Experimental Treatment

Either vehicle (saline or ClinOleic^®^ 20% (Baxter S.L., Valencia, Spain)) or experimental drug treatment were administered through the femoral vein 30 min before the end of the ischemia period. Allopurinol (50 mg/kg) was suspended in saline, and nitroindazole (10 mg/kg) in absolute ethanol and then dissolved in ClinOleic^®^, at a ratio of 1:7.

For this purpose, under 1.5% isoflurane anesthesia, an incision was made in the skin of the inguinal area and the femoral vein was exposed, and the corresponding vehicle or drug was administered using a 27G needle, in accordance with the experimental group (Table 1). To avoid bleeding at the puncture site, direct hemostasis was performed for 1 to 2 min, using a cotton swab. Then, the incision was closed with single stitches.

### 2.3. Tissue Sample Collection and Histological Examination

At the end of the reperfusion period, the animals were anesthetized and 6 cm of terminal ileum (measured from the ileocecal valve) was removed and immersed in tempered saline to gently wash the specimen. Then, the piece was fixed to a plastic guide to keep it stretched and immersed for 24 h in 4% formaldehyde. Each piece of intestine was transversely split into 4 fragments (each 1 cm long), which were embedded in the same paraffin mold. Three paraffin-embedded slices (5 µm) were obtained from each intestine sample (12 sections of intestine from each animal) and stained with hematoxylin/eosin.

The histological injury degree (HID) score was assigned according to the scale summarized in Table 2 (adapted from Chiu et al. [18]). Four quadrants were defined in each histological section and the HID was assigned to each of them. Subsequently, the HID of each section was calculated by summing the index assigned to each quadrant. The HID of each animal was calculated as the mean of the 12 analyzed sections.

### 2.4. Statistical Analyses

All analyses were performed with the GraphPad Prism 6 (GraphPad Software, San Diego, CA, USA), and the minimum significance level was set as *p* < 0.05. The quantitative variables described in this piece of work were represented by the mean and standard deviation. Statistical treatment of the data was performed by analysis of variance (ANOVA). Additionally, once the significant differences between the groups were demonstrated, comparisons of the different groups were performed using Tukey’s multiple comparison test.

## 3. Results

The surgical model used to emulate i-IRI induced the development of i-IRI in 100% of the animals, without resulting in the death of any animal. Overall, the procedure was well tolerated.

**Figure 3:**
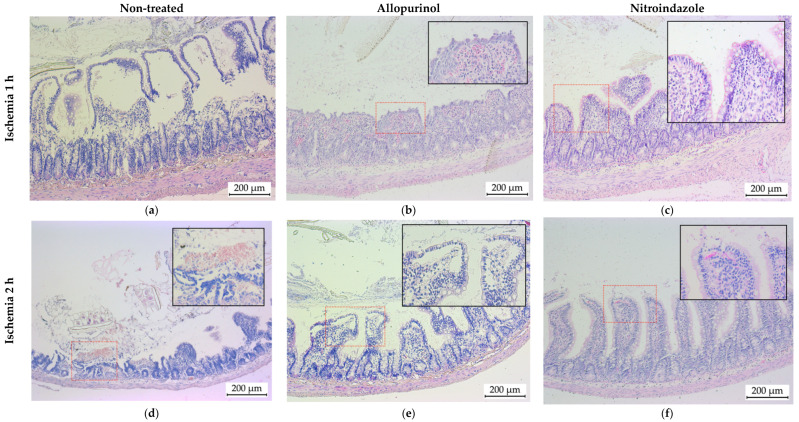
Representative photomicrographs of small intestine sections stained with hematoxylin/eosin. Animals subjected to a period of 1 (**a**–**c**) or 2 h (**d**–**f**) of ischemia, followed by 30 min of reperfusion. The photographs on the left (**a**,**c**) correspond to histological sections obtained from untreated animals, the photographs in the center (**b**,**d**) to animals treated with allopurinol and the photographs on the right (**c**,**f**) to animals treated with nitroindazole.

Histological samples obtained from animals subjected to 1 and 2 h of ischemia showed high intestinal mucosal damage. After 1 h of ischemia and 30 min of reperfusion there was major destruction of the villi (Figure 3a) with the accumulation of inflammatory cells and wide Gruenhagen’s spaces in those villi that had preserved their structure. When doubling the ischemia period (Figure 3d), the damage was more pronounced with the complete loss of the villus structure and fragments of detached villi appearing in the lumen of the intestine. In addition, intraluminal hemorrhage was evidenced by the presence of erythrocytes in the intestinal lumen.

Allopurinol treatment of those animals that underwent 1 h of ischemia resulted in a slight reduction in tissue damage (Figure 3b). Nonetheless, there were evident signs of mucosal injury, such as inflammation (edema of the lamina propria and submucosal layers and presence of inflammatory cells), erythrocyte extravasation and loss of epithelial goblet cells. In the case of 2 h of ischemia (Figure 3e), treatment with allopurinol also resulted in a less severe tissue damage compared to the untreated animals, although extensive epithelial lifting, inflammatory cells presence and edema were still evident.

When nitroindazole treatment was applied, we found a striking improvement in the histological structure of the intestines subjected to ischemia. Thus, after 1 h of ischemia, the structure of intestinal mucosa was almost totally preserved, and the only significant change was the presence of small Gruenhagen’s spaces located at the tip of some villi (Figure 3c). However, nitroindazole was not as effective when administered to animals in which the arterial blood flow of the intestine was occluded for 2 h (Figure 3f). Even though the structure of the villi was better preserved than in the non-treated animals, almost complete loss of the epithelium at the tip of some villi was observed. In addition, evident signs of erythrocyte starvation and inflammatory cell infiltration were found.

**Figure 4:**
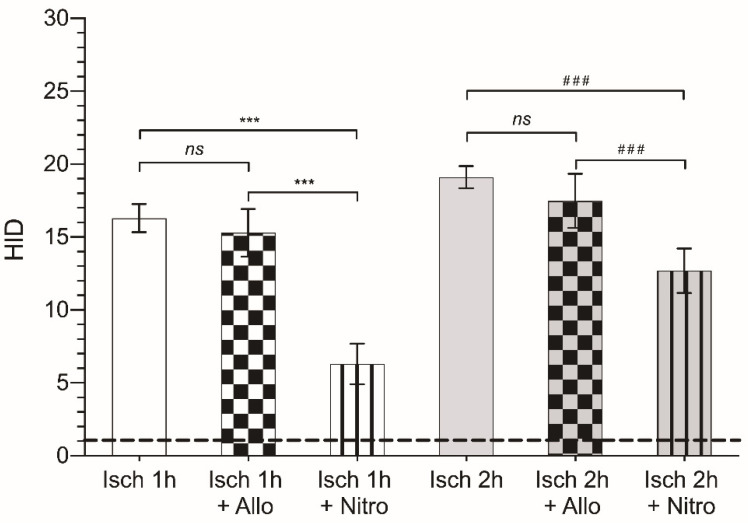
Pharmacological modulation of the histological injury degree (HID) after treatment with allopurinol (grid pattern) or nitroindazole (line pattern), after 1 (white) or 2 h (grey) of ischemia. ***: *p* < 0.001; ^###^: *p* < 0.001; ns: *p* > 0.05. The dashed line indicates the HID score of the control non-ischemic tissue. Non-relevant data (vehicle groups) have been excluded from the figure.

Regarding HID (Figure 4), we observed that the degree of injury after 1 h of ischemia was 15 times higher than that of the control (not subjected to ischemia), 1.06 ± 0.65 vs. 16.3 ± 0.96, respectively (*p* < 0.001). When the ischemia time was doubled to 2 h, although HID did not increase in the same order of magnitude, it was significantly higher (19.10 ± 0.77; *p* < 0.001) than that observed after 1 h. Regarding the vehicles used for the drugs (saline and ClinOleic^®^), neither of them modified the HID (*p* < 0.05; Table 2).

Treatment with allopurinol did not have a beneficial effect on the histological damage induced by 1 h or 2 h of ischemia (15.3 ± 1.64 and 17.49 ± 1.86, respectively; *p* > 0.05). Nevertheless, treatment with nitroindazole did substantially modify the HID observed in the intestinal mucosa. Following 1 h ischemia, HID was reduced by more than half in animals treated with 10 mg/kg nitroindazole (16.3 ± 0.96 vs. 6.3 ± 1.4, *p* > 0.001). In animals subjected to 2 h of ischemia, nitroindazole also induced a significant reduction in HID, however the reduction observed was smaller, decreasing by 1.5-fold (19.10 ± 0.77 vs. 12.69 ± 1.52; *p* < 0.001) (Figure 4 and Table 3).

## 4. Discussion

The effectiveness of antioxidant treatments on IRI has been demonstrated by a large number of previous studies [19,20,21,22,23,24]. Our results are in accordance with this and show that nitroindazole reduces intestinal mucosal damage after i-IRI. SMA clamping is a simple, reproducible procedure with low mortality that allows the simulation of i-IRI for a low output situation (NOMI). In our study, 100% of the animals survived, and we were always able to induce comparable intestinal damage.

We analyzed the relationship between ischemia time and HID, and in contrast to the study by Da Costa et al. [25] where no direct proportionality between both factors was observed, our study shows a significant increase in intestinal mucosal damage as the period of blood flow deprivation increases. These different results could be explained by the type of ischemia induction model that was used; our model recreates a low flow condition when the SMA is clamped, which allows minimal flow because the collateral circulation remains intact. However, in the previously cited model of Da Costa, the aorta is clamped prior to the birth of the renal arteries; in the absence of collateral supply, such a lot of damage occurs in the first minutes of ischemia that it does not change significantly when this period is increased.

As for the relationship between HID and reperfusion time, some studies have shown that the greatest damage, from a biochemical point of view, occurs in the first moments of reperfusion (even in the first 15 min) when those molecules involved in the pathophysiology of i-IRI reach the area deprived of blood flow [24,26,27,28]. In addition, Clark et al. [11] have shown that two 15 min periods of ischemia are more harmful than a period of half an hour. This is explained by the fact that there is a physiological adaptation to time-limited and not very long periods of oxidative stress, which endogenous defense mechanisms are able to cope with [29]. However, if the damage is too severe and prolonged over time, this endogenous system loses its capacity to cope with the oxidative agents [30].

The analysis of the effect of the antioxidant agents tested in this piece of work shows that the prophylactic use of allopurinol during the ischemia period does not achieve a statistically significant reduction in local intestinal damage, regardless of the ischemia time. The literature reviewed showed that several authors have reported results in agreement with those described here. They conclude that when allopurinol is administered, the effect on the prevention of IRI is only significant in those cases in which this treatment is associated with ischemic postconditioning, that is, periods of arterial reocclusion of a few minutes duration applied at the beginning of tissue reperfusion and which have shown a decrease in the damage associated with ROS [31,32]. In contrast, there are also studies in which its oral administration prior to ischemia instauration does demonstrate an antioxidant effect, as evidenced by a reduction in HID and a decrease in the production of oxidative stress markers such as malondialdehyde (MDA) [33]. Other studies performed in a renal IRI mode have shown the beneficial effect of XO inhibition [34,35,36,37,38]. After prophylactic administration of allopurinol, a decrease of up to 75% in the damage was noticed, both biochemical and anatomopathological. In addition to renal IRI models, in studies of this syndrome in upper limbs, allopurinol demonstrated an increase of up to 70% in muscle viability after 6 h of reperfusion [39]. In any case, the pathophysiological mechanism of XO-mediated damage found in rodents and felines, but absent in humans, casts doubt on the already questionable beneficial effect of allopurinol in the management of i-IRI [7].

Regarding the use of nitroindazole in reperfusion injury, most studies have been performed in experimental models of cerebral ischemia. It has been shown that various markers of damage due to oxidative stress, such as MDA, glutathione (GSH) or lipid peroxidation, are greatly reduced with nitroindazole, and reached levels close to those of control groups [27,40,41]. It has been proposed that the prophylactic effect of this compound involves more than its action as an NOS inhibitor, since it is also involved in other enzymatic pathways. Hirabayashi et al. aimed to study the effect of nitroindazole administration on the role of nNOS, in the early phase of cerebral ischemia-reperfusion in mice [42]. Following a 2 h ischemia period and 30 min of reperfusion, they found that nitrotyrosine (NO2-Tyr) formation, a well-known marker for NO-related cytotoxicity, was reduced by 45% and 100% after 25 mg/kg or 50 mg/kg of nitroindazole, respectively. These findings are in accordance to those previously reported by Sorrenti et al. [43] and Liu [44], where nitroindazole increased c-fos mRNA levels, which is related to the recovery of cellular function after cerebral injury. In a murine model of retinal IRI, San Cristobal et al. also reported that prophylactic administration of nitroindazole (10 mg/k) reduced retinal histological damage, the effect being nearly as strong as that achieved by folic acid [45].

It has been proven that the solvent used in the preparation of the nitroindazole solution, ClinOleic^®^ modulates excessive inflammatory reactions [46]. However, in our study we did not found any significant effect in those animals that received ClinOleic^®^ as a transporter for nitroindazole. This emulsion contains 80% refined olive oil and 20% refined soybean oil, and is rich in a large variety of monounsaturated fatty acids (MUFAs) (up to 65%), mainly in the form of oleic acid (C18:1n-9) (found in the olive oil), and lower amounts of polyunsaturated fatty acids (PUFAs) (20%) and triglycerides [47]. According to the United States Food and Drug Administration (FDA), the total amount of both refined olive and soybean oil in ClinOleic^®^ is around 200 g/L [48].

Huang et al. [46] have reported the protective effect of ClinOleic^®^, by decreasing the intensity of the cytokine storm and apoptosis in an acute lung injury model. These results may seem opposed to those found in our work, however, we must take into account that in their study ClinOleic doses of 6 g/kg/day were administered for 7 consecutive days before the induction of lung damage (total dosage over 10 mL), while we administered a single dose of 0.43 mL of ClinOleic^®^.

Furthermore, the concentration of the antioxidants included in ClinOleic^®^ are quite different from those used in therapeutic studies. For example, Farías et al. [49] used 225 g rats that received daily doses of 135 mg of PUFAs over 8 weeks, while our animals received just one dose of 13.76 mg of PUFAs. A similar disparity can be observed with the experiments of Sukhotnik et al. [50].

ClinOleic^®^ also has tiny quantities of α-tocopherol, an active isomer of vitamin E with high antioxidant capacity [47]. Its presence could explain the better results obtained in animals treated with nitroindazole since it acts at different levels and in a synergistic manner. However, the total amount of α-tocopherol administered to each animal was below 0.04 mg/kg. This amount is more than 500 times lower than the dose of this compound administered when used for therapeutic purposes [51,52,53], so the effect of the α-tocopherol contained in ClinOleic^®^ would be minimal. In any case, no benefit was observed when it was administered alone, without nitroindazole. Moreover, when α-tocopherol was tested in several models of IRI, it was shown that it has an enhancing effect over other treatments, such as allopurinol [54], vitamin C [28], or ischemic preconditioning [55]. However, Catilayan et al. [19] studied its isolated effect on i-IRI and found no statistically significant differences compared to the non-treated groups.

## 5. Conclusions

In our i-IRI experimental setting, we observed that the duration of the ischemia period has a directly proportional effect on intestinal mucosa damage. The use of allopurinol achieved a discrete but not significant reduction in intestinal damage, ruling out its applicability in this model of i-IRI. On the other hand, nitroindazole administered prophylactically during ischemia significantly reduced the histological injury, with its effect being more striking when ischemia was limited to 1 h.

## Figures and Tables

**Table 1:** Experimental groups.

Group	Ischemia	Reperfusion	Treatment	N° of Animals
0	no	no	no	6
1	1 h	30′	no	6
2			Saline	6
3			ClinOleic^®^ 20% ^1^	6
4			Allopurinol	6
5			Nitroindazole	6
6	2 h	30′	no	6
7			Saline	6
8			ClinOleic^®^ 20% ^1^	6
9			Allopurinol	6
10			Nitroindazole	6

^1^ ClinOleic^®^ 20% (Baxter S.L., Valencia, Spain).

**Table 2:** Criteria to assess the corresponding histological injury degree (HID), according to Chiu et al. [18].

Grade	Description
0	Normal mucosal villi; no histological changes
1	Epithelium of the villi is almost fully preserved; development of Gruenhagen’s subepithelial spaces (normally located in the apex); capillary congestion
2	Extension of the subepithelial spaces with moderate lifting of the epithelial layer of the lamina propria
3	Preserved villous structure with almost complete loss of epithelium (preservation > 50%); presence of intraluminal hemorrhage.
4	Destructuring of the villi, mostly denuded (preservation < 50%)
5	Loss of villi, disintegration of the lamina propria; hemorrhage and ulceration

**Table 3:** Histological injury degree (HID) following a period of ischemia of 1 or 2 h and 30 min of reperfusion in control- or treated-animals (vehicles, allopurinol or nitroindazole). The statistical significance shown corresponds to the HID of non-treated animals subjected to i-IRI.

Group	Control	Saline	Allopurinol	ClinOleic^®^	Nitroindazole
Ischemia 1 h	16.3 ± 0.96	15.2 ± 1.91 *ns* ^1^	15.3 ± 1.64 *ns* ^1^	14.4 ± 0.98 *ns* ^1^	6.3 ± 1.4 *p* < 0.001
Ischemia 2 h	19.10 ± 0.77	18.1 ± 0.9 *ns* ^1^	17.49 ± 1.86 *ns* ^1^	17.9 ± 0.67 *ns* ^1^	12.69 ± 1.52 *p* < 0.001

^1^ *ns*: not significant (*p* > 0.05).

## Data Availability

The data that support the findings of this study are available from the corresponding author, (B.H.P.), upon reasonable request.
